# Cross-cultural validation and psychometrics’ evaluation of women’s experience of maternity care scale in French: the ESEM

**DOI:** 10.1186/s12874-020-01052-5

**Published:** 2020-07-11

**Authors:** L. Floris, C. de Labrusse

**Affiliations:** 1 HESAV School of Health Sciences, HES-SO University of Applied Sciences and Arts Western Switzerland, 1011 Lausanne, Switzerland; 2 University of Geneva, 1211 Geneva, Switzerland

**Keywords:** Women’s satisfaction, Maternity services, Hospital, Midwifery, Comprehensive support, Questionnaire, Psychometric evaluation, Scale, Cross-cultural validation

## Abstract

**Background:**

Evaluating women’s satisfaction should reflect the entire maternity care experience (antenatal, intrapartum and postnatal). The Women’s Experience Maternity Care Scale (WEMCS) questionnaire enables this assessment. The purpose of this study was to translate in French, adapt and explore the psychometric properties of the WEMCS and to determine the best cut-off on the optimal satisfaction for the three scales.

**Methods:**

Backward, forward translation and cross-cultural adaptation were processed to validate the French version of WEMCS: Échelle de Satisfaction de l’Experience des soins en Maternité (ESEM). Psychometric tests assessed the questionnaire, which includes three scales, such as construct validity, internal consistency, Cronbach’s alpha coefficients and ceiling and floor effects. A receiver operating characteristic (ROC) curve was used to determine the best cut-off values for optimal satisfaction. Reproducibility was verified by test–retest reliability.

**Results:**

Primiparas with uncomplicated pregnancies were recruited antenatally at the University Hospital of Geneva. Of the 229 patients who agreed to participate, 202 women (88.2%) returned the test and retest questionnaires. Principal component analysis for the antenatal, intrapartum and postnatal scales suggested the unidimensional character of the three scales; Cronbach’s alpha coefficients were high for the three scales with values of > 0.85. Construct validity based on the five-point Likert scale values showed a Spearman’s rho correlation of *r* = 0.56 for the antenatal scale (*p* < 0.001) and *r* = 0.62 for the intrapartum scale (*p* < 0.001), as well as a strong correlation with the postnatal scale, with *r* = 0.78 (*p* < 0.001). Optimum cut-off scores for the ROC curve of the antenatal, intrapartum and postnatal scores were equal to or higher than 48, 50 and 70, respectively. The three scales showed good sensitivity and good specificity. The stability of the ESEM questionnaire was confirmed by intra-class correlation coefficients of > 0.80. However, the three scales revealed ceiling effects.

**Conclusion:**

The psychometric proprieties of the ESEM demonstrate it’s ability to evaluate the quality of perinatal health care. The ESEM should be tested in the context of different models of women’s care and with women with different degrees of pregnancy complications to explore the validity of this scale.

## Background

Reliable quality measurements of care in maternity services are essential for improving the delivery of care [1]. According to the literature [2, 3] potential benefits may be achieved by integrating several parameters in health services organisation, such as safety, efficacy, efficiency and equity [4]. Switzerland, specially, shares this view, as one of its hospitals in the western part of the country has a goal to ensure high care quality in all aspects of its activity [5]. Among the outcomes used to measure the improvement of health care, satisfaction remains one of the most frequently reported [1, 6–8]. Women’s satisfaction with maternity care has been increasingly reported in the literature but is inconsistently addressed as either an overall experience of maternity care or a focus on childbirth, rather than observing distinct episodes of women’s experiences [8–10]. Maternity care is provided throughout the entire perinatal period and encompasses three main phases: the antenatal, intrapartum and postnatal periods [11]. Three distinctive measurements of satisfaction are essential, because, for some women, care during the antenatal period will have an impact on satisfaction measures in the childbirth and postpartum periods. Additionally, with the unpredictability of childbirth, differentiating between perinatal periods could help disentangle specific episodes from the general experience of care to clarify women’s experiences and related satisfaction/dissatisfaction [12]. A tool, which integrates the three main stages of care, is therefore essential. Scales are available to measure the expectations of control or experience of the patient and her environment during childbirth, such as the Labour Agentry Scale (LAS) [13], the Birth Satisfaction Scale (BSC) [14], the Childbirth Experiences Questionnaire (CEQ) [15], the Women’s Views of Birth Labour Satisfaction Questionnaire (WOMBLSQ) [16] and the Edinburgh Postnatal Depression Scale (EPDS) [17]. These scales only measure some specific aspects of perinatal care. Other scales have been developed but were presented in studies with limited methodology [18], such as stability and reliability and they have all failed to provide an evaluation of the overall maternity care experience [19]. Furthermore, given that several satisfaction scales exist to evaluate the maternity experience, there is a lack of available satisfaction scales in French for collecting data regarding specific moments of maternity care.

The ‘Women’s Experience of Maternity Care’ (WEMCS) scale has been used extensively in the literature and offers the ability to discriminate between personalised aspects of care and the periods of perinatality which have been identified in the literature to have an impact on satisfaction [20–23]. The original English questionnaire was the result of various iterations of change. The first questionnaire, which was developed by Brown et al. in 1994, was based on the works of Cartwright (1986–1988) [9]. The aim of this first version was to investigate the experience of satisfaction with different models of care during antenatal and intrapartum care with overall ratings, specific questions on care satisfaction and open questions on several aspects of care. The ratings were dichotomised (satisfied/ dissatisfied) and were adjusted for specific factors or evaluated based on aspects of women’s care; 790 Australian women were included in this study. In 1997, Brown et al. improved the questionnaire based on the experience of 1994 and conducted various tests of this new questionnaire with women. The form of statements on the quality of antenatal, intrapartum and postnatal care were denoted with five or six levels of appreciation. The development of different characteristics of satisfaction and experience of childbirth were illuminated with this publication. The intent was to develop questions to consider some specific qualitative aspects of care (Example – Active say in decision making: always, mostly, sometimes, rarely, not at all and not sure). In addition, three questions were added to consider overall satisfaction/dissatisfaction regarding the periods of care (antenatal, intrapartum and postpartum); the options for this answer were: very good, good, mixed poor and very poor. This version was tested in large populations with Australian women (*n* = 1336) and in Sweden in 1994, with 1230 women in the Randomized Controlled Trial (Experience of Childbirth in Birth Centre) [24]. A few years later, Waldenström (1999) adapted this version to consider the experience of birth, in the Swedish context, with 1111 women [25]. Both the statement and the scoring of the questions were changed using a Likert scale with seven points to consider each aspect of care separately. Based on the consideration of specific aspects of quality of care, the result is a measurement of the consideration of 30 statements in three sections (scales). Although used in various studies, including in the study COSMOS [22], this questionnaire, ‘Women’s Experience of Maternity Care’ (WEMCS), was not submitted to psychometric testing or to the process of validation in the original language and was not adapted to languages other than English.

Beaton, Bombardier, Guillemin and Bosi Ferraz (2000) described ‘cross-cultural adaptation’ as follows: ‘to encompass a process which looks at both language (translation) and cultural adaptation issues in the process of preparing a questionnaire for use in another setting’ (p. 1) [26]. With 47% of births in Geneva involving women from EU and non-EU countries outside of Switzerland, the translation and cultural adaptation of the questionnaire were essential while taking into account the cultural diversity of these women to ensure the content validity of the tool [3]. Following Beaton et al. (2000) steps of translation and cultural adaptation would ensure that the questionnaire reflected and evaluated experiences in a similar way across a multinational sample of women.

In the present study, the latest version of the WEMCS questionnaire was chosen for French cultural adaptation and translation with an evaluation of the psychometrics properties [20, 27]. After the translation of the scale, it was essential to test the properties – such as internal consistency, reproducibility, validity and responsiveness of the French version of the instrument. In addition, in order to assess the potential of the measure of satisfaction for each scale, we proposed to test the score of each scale individually to differentiate between satisfaction and dissatisfaction. The WEMCS measures separately specific items on women’s antenatal, intrapartum and postnatal experiences of care on the Likert scale of 1 to 7, corresponding to 1 = strongly disagree through 7 = strongly agree. The number and the percentage of each statement were evaluated separately. Similarly to the original questionnaire, to differentiate the level of satisfaction. The seven-point rating of each statement was dichotomised with optimal satisfaction versus non-optimal satisfaction, with the assumption that the aggregated number and the percentage of ratings 6–7 were considered as optimal satisfaction, while less optimal satisfaction was considered with the number and the percentage of ratings 1–5 [20, 25]. This cut-off (1–5 versus 6–7) was retained by the authors because, after various tests, it did not alter the major variables of the study [25]. The appraisal of the quality of the statistical analysis of specific aspects of care is essential, as explained by Brown et al. [9], therefore these data could be optimised and used for the global measurements of each scale, using the global results of 8, 10 and 12 statements for each period of care, such as antenatal, intrapartum and postnatal. Additionally, in order to identify mothers with optimal satisfaction or non-optimal satisfaction, each score can be dichotomised using best cut-off values for the three scales.

These properties provide important information on the value of this instrument in relation to potential use. With these hypotheses, the WEMCS instrument can accurately evaluate women’s satisfaction as an indicator of quality for all periods of maternity care.

This paper aims to:

- Describe a process of translation and cross-cultural adaptation of the WEMCS in a French context.
- Explore the psychometric property of the French translation of the WEMCS (ESEM), including the identification of the best cut-off on the optimal satisfaction for the three scales of the ESEM.

## Methods

### Material and procedures

The Women’s Experience of Maternity Care Scale (Additional file 1), was originally written in English and includes 30 quantitative items divided into three sections (scales) subjected to a process of evaluation: (1) antenatal care (8 items), (2) intrapartum care (10 items) and (3) postnatal care (12 items). A seven-point Likert scale ranging from 1 to 7 and corresponding to 1 = strongly disagree through 7 = strongly agree allows for a detailed assessment of each item [20]. The statements are phrased positively, with one exception: ‘I often felt the doctors/midwives were very rushed’. An item related to an overall assessment was provided at the end of each section, such as ‘On balance, how would you describe your care?’, using the same seven-point Likert scale (1 = very poor, 7 = very good) to allow a global evaluation of each period with one item [25]. The translated questionnaire was named ‘Échelle de Satisfaction de l’Experience des Soins en Maternité, or ESEM (Additional file 2).

### Translation and cultural adaptation

The process of translation and cross-cultural adaptation from the English version to a French version was conducted through a precise, rigorous method according to recent guidelines using the five steps of translation and cross-cultural adaptation developed by Beaton, Bombardier, Guillemin and Bosi Ferraz (2000). These include translation, synthesis, back translation, expert committee and pretesting. The process occurred in this study as follows: (1) Translation. After approval was obtained from the authors [20, 22], three professional translators performed three independent translations; (2) Synthesis. From the three translations, a panel of experts (a midwife, clinical manager, researcher and lecturer) reached a consensus on each question; (3) Back translation. Back translation into the original language was performed by two (blinded) professional translators (specialists in the field of medical translation); and (4) The fourth and fifth steps − evaluation by an expert committee and pretesting − were conducted simultaneously.

Problematic issues were addressed, then adapted by the panel of experts, who revised the translation, helped address the different perspectives and amended the questionnaires accordingly, finally the specific items were pre-tested again. Recent guideline from Perneger et al. (2015) suggested the number of pre-tests should be determined based on the collected results and the prevalence of the problem encountered in the cognitive interview. Therefore, 10 to 30 participants could be necessary for the pre-tests. In this study, 15 women with the same characteristics as the target population and who met the inclusion criteria were required to participate in the pre-testing [28]. These women were not included in the sample. The pre-test was conducted through individual interviews (cognitive interviewing) with the support of a specific grid. The support grid was used to explore the comprehension of the questionnaire. It was based on the publication of Sousa et al. [29] and conducted with the panel of experts. The questions were detailed according to the section (antenatal, intrapartum and postnatal). The form and the clarity of the terms used in the questionnaire were evaluated. The women were invited to comment on the clarity of the instructions and the precision of the questions (using dichotomous consideration), after which they were invited to explain each question with their own words. Their opinions were discussed, along with the points attributed on the Likert scale. The interviews proceeded, by phone, between one and 2 weeks after the return of the questionnaire.

### Translation process

Some of the questions translated into French included additional words compared with the English questions to ensure that women would understand the topic raised. See the following examples:

Item from the Women’s Experiences Maternity Care Scale: ‘I was happy with the emotional support I received in pregnancy from doctors/midwives’.

From translator 1: ‘J’ai été satisfaite du soutien moral reçu de la part des médecins/sages-femmes durant ma grossesse’.

From translator 2: ‘J’étais satisfaite par le soutien émotionnel dont j’ai bénéficié de la part des médecins/sages-femmes lors de ma grossesse’.

From translator 3: ‘J’ai été satisfaite du soutien psychologique reçu de la part des médecins/sages-femmes pendant ma grossesse’.

The consensus of the team was ‘J’ai été satisfaite du soutien moral (émotionnel) reçu de la part des médecins et des sages-femmes pendant ma grossesse’.

In back-translation, this became ‘I was satisfied with the moral (emotional) support received from doctors and midwives during my pregnancy’.

We identified some difficulties for women in answering the question regarding the care provided by midwives versus that provided by doctors. An explanation may be a lack of differentiation between the role definition of the professional and the care that he/she should provide. This can be reflected in the difficulty to confidently complete questions regarding the care provided by a midwife versus the care provided by a doctor. As an illustration, one woman stated: ‘The doctor and midwives were very rushed’ on the prenatal, intrapartum and postnatal scales, but, conversely, she responded positively on the other questions in the rest of the questionnaire. This perhaps addresses an issue of translation or incorrect wording of the question, which can decrease the validity of the answers from the same woman across time.

### Exploratory factor

Construct validity of the ESEM was tested with exploratory factor analysis (EFA) [30]. Factor analysis was used to investigate how the observed variables were associated with latent constructs. Principal component analysis (PCA) was used to identify the number of dimensions of the three scales and to examine the underlying structure of the questionnaire. The sample adequacy was tested with Bartlett’s test of sphericity based on the criteria of *p* < 0.001 and with the Kaiser-Meyer-Olkin (KMO) test based on a criterion of ≥0.7 [31]. Principal components were identified with the Kaiser’s criterion of eigenvalues of 1, an examination of the Cattell scree plot and with the percentage of variance explained. Finally, parallel analyses were done to confirm the number of components retained [32]. To identify the factor structure of each scale, we explored the interrelation between the item and the factors. The statistical measures reported from the analysis included communalities and component loadings. Factor loading of 0.30 were considered the minimum acceptable, items of at least 0.45 were considered fair, those of at least 0.55 were considered good, those of at least 0.63 were considered very good and those greater than 0.71 were considered excellent [33]. Communalities (sum of the squared factor loadings for each variable) were used to determine how each variable is explained by the factors. Values ≥0.40 were considered acceptable values [34].

### Responsiveness

The sum of each scale of the French version of ‘Women’s Experience of Maternity Care’ (ESEM) was compared with the five-item scale values: bad, poor, good, very good and excellent, using the Spearman’s rho correlation. A correlation above 0.68 ensured construct validity [35]. A Receiver Operating Characteristic (ROC) curve was used to determine the best cut-off values for optimal satisfaction or non-optimal satisfaction on the three scales. Sensitivity and specificity were calculated based on cut-off values using the Youden index and the area under curve (AUC) to indicate the relevance of the results, such as the probability that levels of satisfaction were appropriately identified. In the absence of a gold standard measure, the five-item Likert scale (bad, poor, good, very good, excellent), dichotomised with excellent and very good versus bad, poor and good during antenatal, intrapartum and postnatal periods, was used to discriminate between optimal satisfaction and non-optimal satisfaction. This measure and this method of discrimination were based on a general institutional survey of the global quality of health care, used to determinate the problematic values coding algorithm by Picker Institute Europe [36].

The consistency of the questionnaire was determined based on the stability of this tool over time. The association between the satisfaction score of the three scales and how much time elapsed between giving birth and completing the return questionnaire was tested.

### Internal consistency

The reliability of the instrument was estimated using internal consistency by calculating Cronbach’s alpha to test the interrelatedness among the items of each scale (antenatal, intrapartum, postnatal) [37]. A Cronbach’s alpha of between 0.7 and 0.9 was expected [38].

The homogeneity of the interrelated items for each of the three scales was measured by Spearman’s rho correlation. To assess the degree of consistency between the components of each level, the sum of the items and the overall satisfaction score (without the item) were assessed using Spearman’s correlation coefficient. Values of *r* ≤ 0.35 were considered to be a weak correlation, 0.36 to 67 a moderate correlation and above 0.68 a strong correlation [35]. In this study, strong correlations using these tests were attempted.

### Floor and ceiling effects

Floor and ceiling effects were tested for the three scales. The greater of 15% of high or low values were considered limited results of the questionnaire and may indicate insufficient scale performance [37].

### Reproducibility

The time period between the test and the retest was 1 month [37]. This period of time was selected to prevent recall bias and with consideration for the responsibilities of a new mother and the potential difficulty in sending the questionnaire immediately after childbirth. This element was consistent with a previous study [39].

### Reliability

The relative reliability was calculated for each scale using intraclass correlation coefficients (ICC) and confidence intervals. The ICC measures variability between the individual results and the error of measurement coefficients; a minimum of > 0.70 was attempted [40, 41].

The standard error of measurement (SEM) and SEM percentage allows for the evaluation of the absolute retest reliability [40]. The SEM was used to evaluate the precision of the instrument between two assessments in each scale. The smallest real difference (SRD) and the SRD percentage between the two measures were also estimated in each scale [42]. In this sample, the smallest change was sought [37].

### Agreement

The Bland-Altman plot was used to measure the limit of agreement. Standard errors of measurement and the proportion of agreement were used to check the stability of each scale in the instrument over time and to assess the acceptable fluctuation limit [41, 43]. To ensure the stability of the instrument, a minimum number of outliers should be used.

### Acceptability

To evaluate the acceptability of the ESEM, we examined the return rate and the time between sending and receiving the returned questionnaire. The amount of missing data and the distribution of responses were assessed. The missing data for each scale were listed. If one or two items were not completed, they were replaced by the average divided by the number of items completed. If more than two items were not completed, the section was not included in the analysis.

### Convergent validity

Several measures are required to demonstrate convergent validity. In this study, this test could not be effectively conducted because of a lack of similarly validated French scales in the area of perinatal care that evaluate satisfaction antenatally during labour and postpartum [18].

### Independent variables

The demographic data included the age of the participant, place of birth, level of education, marital status, occupation, annual family income and tobacco use. Obstetrical outcomes were extracted from the medical records and included the gestational age, parity, mode of delivery, labour induction and analgesia.

### Statistical analysis

Descriptive analyses of the scores by scale were performed (the minimum and the maximum scores, the mean and standard deviation or the interquartile range). The process to examine the psychometric properties was performed with a continuous score. The data were tested using the Kolmogorov-Smirnov test to verify the normality of the distribution. The number and the percentages of obstetric and neonatal issues are presented. The linear regression was used to estimate the associations between the time taken to return the questionnaire and the score of satisfaction on the three scales.

As mentioned above, internal consistency was evaluated with Cronbach’s alpha. The homogeneity of interrelated items was measured using the Spearman’s rho correlation coefficient. The reliability coefficient of the result was calculated for each scale using the ICC with a 95% confidence interval (CI). The statistical significance level was considered *p* < 0.05. The means of the difference were presented using the Bland-Altman plot method and the standard error of measurement (SEM = SD diff / √2) [42].

### Sample size

There is no valid a priori scientific recommendation for determining the adequacy of a sample size which has been recommended in guidelines for validating measurement scales with one principal parameter such as factor analysis [44–46]. Most studies have employed empirical strategies for estimating an adequate sample size, such as a ratio of the number of items by subject - e.g., 1:5 or 1:10 [47] - or a classification system that appraises items into five classifications, ranging from 100 subjects (poor) to 1000 subjects (excellent) [45, 46]. The theoretical framework defined by MacCallum for estimating sample size can be confirmed only a posteriori. In this framework, sample sizes are independent of the number of survey items. MacCallum suggests that, based on Monte Carlo simulation, the quality of the data could be used to determine an adequate sample size. In his theory, the quality of the data is ascertained by the degree of variation between the variables, such as assessing the level of communality (the part of the variance explained by common factors) [48].

In the present study, the analysed results included a strong a posteriori factor loading (over 0.7) and high communalities (over 0.6) for most variables. These results show that a sample of *N* = 200 would be sufficient to explore our research question and psychometric properties, which supports MacCallum’s hypothesis for estimating sample size [48].

### Sample

Participants were recruited in a waiting room before the antenatal consultation or during their first or second antenatal consultation at the University Hospital of Geneva (HUG)*.* The enrolments were carried out by both the research team and the hospital midwives on workdays after verifying the eligibility criteria. The convenience sampling method was used in the enrolment. This maternity hospital is the largest public maternity facility in the French part of Switzerland, with 4000 births annually. Eligible women had low-risk pregnancies, according to the institutional norms, including no pathological obstetric history, such as preeclampsia, intrauterine growth restriction, preterm birth or miscarriage. In addition, eligible women did not have pathological medical conditions, such as hypertension, diabetes, renal disease, thrombocytopenia, hemoglobinopathy or psychiatric disorders, and they needed to have a stable psychosocial situation, to be able to read French and to have a predicted birth in this hospital. The women who agreed to participate completed a written consent form and provided socioeconomic data during the antenatal consultation. To measure the quality of global satisfaction of care received during the three periods – antenatal, intrapartum and postnatal – a five-item Likert scale (bad, poor, good, very good, excellent) was part of a general institutional survey and completed separately to the ESEM. These measurements were previously used on the general institutional survey.

### Data collection

The data collection was processed on different forms. The first part was completed by the women at the same time that they enrolled and signed the consent form. This first part was in paper format, and the questions involved socio-demographic data. The obstetrical data were extracted from the patients’ files. Two months after childbirth, the women received the questionnaire, ESEM, in paper format, by post (at their homes). They could send the questionnaire back in the enclosed return (pre-paid self-addressed) envelope. This method was chosen because not all mothers were using the internet in 2013–2014 and there are some social disparities in Switzerland concerning internet access. If participants did not initially respond, they were called by phone and subsequently mailed a letter to improve response rates. The questionnaire was sent 2 months after birth in accordance with the methodology used by others authors [24–26]. Delay of publication occurred because authors’ working agendas, editorial and peer-review processes.

### Data processing

The results were analysed using SPSS version 22, except the exploratory factor analysis, the Receiver Operating Characteristic and the figures, which were produced using Stata 12.

### Ethical framework

The information provided was collected after patient consent forms were obtained during the antenatal consultation by both the midwives and research staff. This protocol was approved by the Departmental Ethics Committee of Maternity-Paediatrics, HUG, and is registered under CER 11–242.

## Results

### Sample and recruitment procedure

Patients were recruited in two periods (14 May 2012 to 30 November 2012 and 17 June 2013 to 28 August 2013) both by the midwives and by research staff. The enrolments were conducted either before or during an antenatal visit at the maternity department. At inclusion, the mean of gestational weeks was 34.1 (SD = 8.7); the median (50th percentile) was 37.0 and the 25th and 75th percentiles were 32.8 and 40.0, respectively. Maternal age was a mean of 31.5 years (SD = 4.4). The median (50th percentile) was 32.0, and the 25th and 75th percentiles were 28.0 and 34.0, respectively. Additional patient characteristics are given in Table 1.

**Table 1:** Participant characteristics *n* = 202

	*n* (%)
**Maternal age in years,** mean ± [SD]	31.5 [4.4]
**Gestation in weeks,** mean ± [SD]	39.9 [1.0]
**Nulliparous women**	125 (61.9)
**Place of birth**	
Swiss	87 (43.1)
European	77 (38.1)
Non-European	38 (18.8)
**Education**	
Mandatory, internship	48 (23.7)
College diploma	38 (18.9)
University	116 (57.4)
**Marital status**	
Married	134 (66.3)
Living with a partner	59 (29.2)
Other	9 (4.5)
**Occupation**^**a**^	
Stable job	153 (75.7)
Other	27 (13.4)
Unemployed	15 (7.7)
**Annually family income**^**b**^	
≤ 100′000 CHF	94 (46.5)
> 100′000 CHF	70 (36.7)
**Tobacco use**^**c**^	
Never smoked	104 (51.8)
Stopped smoking	45 (22.4)
Smoked before pregnancy	52 (25.8)
**Mode of delivery**	
Spontaneous	140 (69.3)
Assisted vaginal	33 (16.3)
Caesarean section	29 (14.4)
**Induction**	
No	126 (54.5)
Yes	76 (45.5)
**Analgesia**^**d**^	
Not at all	53 (30.6)
Epidural	120 (69.3)

^a^ Missing values, *n* = 7

^b^ Missing values, *n* = 38

^c^ Missing value, *n* = 1

^d^ Without Caesarean section

### Acceptability

**Fig. 1:**
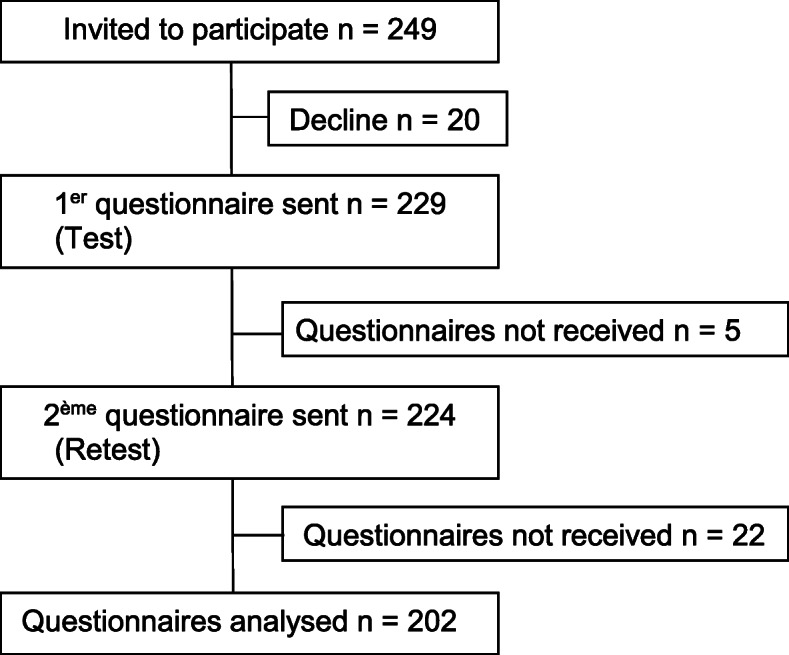
Study flowchart

Of the 229 women who agreed to participate to processed of psychometrics’ evaluation of the ESEM, 202 (88.2%) of them returned both questionnaires. Details are in Fig. 1. Three attempts at contact were made by mail or by phone to increase the response rate. The mean of days between giving birth and the return of the questionnaire was 84.5 days (SD = 22.8).

In the sample, 19 questionnaires contained missing data. In accordance with the methodology, the antenatal scale results for six questionnaires were not included in the analysis because there were more than two missing values. For three questionnaires with isolated values missing from the antenatal scale, these values were replaced with the mean score of the scale. Three isolated missing values were replaced with the mean score for the intrapartum scale, as well. For the postnatal scale, seven questionnaires had one or two missing values, which were replaced with the mean score.

### ESEM descriptive analysis

The mean and SD of the antenatal scale was 49.6 (6.6), median 51.0 [interquartile range (IQR) 46.0–55.0-1.2], for the intrapartum 64.2 (8.2), median 67.0 [interquartile range (IQR) 61.0–70.0], and for the postnatal 70.4 (14.2), median 74.0 [interquartile range (IQR) 65.0–72.0]. Floor effects of 1% and ceiling effects of between 16.8 and 33.2% occurred. For the three scales, the normal distribution using the Kolmogorov-Smirnov Z-test was *p* < 0.001. The other details of the descriptive data are shown in Table 2.

**Table 2:** Properties of the ESSIC scales

	Antenatal *n* = 196	Intrapartum *n* = 202	Postnatal *n* = 202
Nb Items	8	10	12
Scales min-max	8–56	10–70	12–84
Mean (SD)	49.6 (6.6)	64.2 (8.2)	70.4 (14.2)
Cut-off ≥	48	60	70
Optimal satisfaction (nb%)	145 (74.0)	174 (86.1)	132 (65.3)
Non-optimal satisfaction (nb%)	51 (26.0)	28 (13.9)	70 (34.7)
Sensitivity %	83.2	91.0	82.5
Specificity %	63.0	83.3	84.4
AUC (CI 95%)	0.80 (0.73–0.85)	0.92 (0.87–0.95)	0.88 (0.83–0.93)

*AUC* Area under the curve

### Exploratory factor analysis

The three scales showed an adequacy and a suitability for use in performing an exploratory analysis with a KMO value > 0.80, and the Bartlett’s test of sphericity was statistically significant (*p* <. 001). Principal component analysis for the antenatal, intrapartum and postnatal scales shows only one component with Eigenvalue > 1, explaining 54.93, 61.87 and 70.30% of the variance, respectively. These results were confirmed with a scree plot and with the parallel analysis suggesting one dominant factor and underlining the unidimensional character of the three scales. The detail of this result is shown in Table 3. The exploratory factor analysis showed strong factor loading for the antenatal, intrapartum and postnatal item scale of > 0.70, except for the item, ‘I often felt the doctors/midwives were very rushed’, which showed a lower factor for the antenatal and intrapartum scales (factor loading ≤0.50). The proportion of variability of each variable which is explained by the factors, described with the communalities, show satisfactory results, with the result ≥40, except for the item, ‘I often felt the doctors/midwives were very rushed’, on the antenatal and intrapartum scale, which presented low communalities < 40 (Tables 4, 5, and 6).

**Table 3:** Factor analysis of ESEM scales

Scales	Items	Factors > 1	Eigenvalue	Parallel Analysis^a^	Eigenvalue adjusted	Variance Explained (%)	KMO	Bartlett
Antenatal (*n* = 196)	8	1	4.39	1	4.12	53.55	0.89	<.001
Intrapartum (*n* = 202)	10	1	6.19	1	5.74	61.87	0.91	<.001
Postnatal (*n* = 202)	12	1	8.01	1	8.45	70.43	0.94	<.001

^a^Number of factors

**Table 4:** Exploratory items factor analysis of antenatal scale of ESEM

Items	Mean (SD)	Loading of PCA	Communalities
1. At my check-ups I was always asked whether I had any questions.	6.46 (1.09)	0.72	0.51
2. Often at my check-ups the doctors or midwives were very rushed.	5.38 (1.81)	0.53	0.29
3. I always felt my worries, anxieties or concerns about the pregnancy and the baby were taken seriously by the doctors/midwives.	6.16 (1.27)	0.70	0.47
4. I was always kept informed about what was happening and doctors/midwives made an effort to explain anything I didn’t understand.	6.32 (1.10)	0.77	0.60
5. I was happy with the physical care I received in pregnancy from doctors/midwives.	6.41 (0.86)	0.79	0.62
6. I was happy with the emotional support I received in pregnancy from doctors/midwives.	6.27 (1.16)	0.82	0.67
7. I was always given an active say in decisions about my care in pregnancy.	6.28 (1.10)	0.73	0.53
8. On balance, how would you describe your CARE during pregnancy?	6.33 (0.80)	0.84	0.70

**Table 5:** Exploratory items factor analysis of intrapartum scale of the ESEM

Items	Mean (SD)	Loading of PCA	Communalities
1. The midwives and doctors always kept me informed about what was happening and made an effort to explain anything I didn’t understand.	6.50 (0.94)	0.82	0.68
2. I was always given an active say in decisions about care during labour and birth.	6.34 (1.03)	0.74	0.54
3. The doctors/midwives were sensitive and understanding.	6.57 (0.89)	0.90	0.80
4. The doctors/midwives were encouraging and reassuring.	6.64 (0.94)	0.89	0.80
5. I often felt the doctors/midwives were very rushed.	5.63 (1.94)	0.49	0.24
6. Care during labour and birth was provided in a safe and competent way.	6.56 (0.99)	0.71	0.50
7. I was happy with the physical aspect of care by doctors/midwives.	6.46 (1.06)	0.77	0.60
8. I was happy with the emotional support I received by doctors/midwives	6.48 (1.13)	0.85	0.72
9. My needs of privacy were well respected during the labour and birth	6.52 (0.95)	0.70	0.49
10. On balance how would you describe your CARE in labour in labour and birth?	6.47 (0.94)	0.91	0.83

**Table 6:** Exploratory items factor analysis of postnatal scale of the ESEM

Items	Mean (SD)	Loading of PCA	Communalities
1. I was always kept informed about what was happening, and doctors/midwives made an effort to explain anything I didn’t understand.	5.97 (1.49)	0.83	0.70
2. I was always given an active say in decisions about the care of my baby and myself.	5.99 (1.26)	0.70	0.49
3. I was given the advice and support I needed in how to handle, settle or look after the baby.	5.67 (1.59)	0.87	0.76
4. I was given the advice and support I needed in any problems with the baby’s health and progress.	5.90 (1.43)	0.88	0.78
5. I was given the advice and support I needed about my own health and recovery.	5.93 (1.42)	0.89	0.80
6. The midwives/doctors were sensitive and understanding.	5.96 (1.39)	0.91	0.83
7. The doctors/midwives were encouraging and reassuring.	6.06 (1.36)	0.93	0.86
8. I often felt the doctors/midwives were very rushed.	4.57 (2.19)	0.64	0.40
9. Care in hospital after the birth was provided in a safe and competent way.	6.30 (1.10)	0.81	0.65
10. I was happy with the physical aspect of care by doctors/midwives.	6.26 (1.10)	0.82	0.66
11. I was happy with the emotional support I received by doctors/midwives.	5.77 (1.60)	0.91	0.82
12. Thinking back now, how would you describe the care you and your baby received in hospital after the birth?	6.02 (1.78)	0.84	0.71

### Validity

Each scale was significantly correlated with the total score of the five-item Likert scale values. The Spearman’s rho correlation for the total score was *r* = 0.56, for the antenatal scale (*p* < 0.001), *r* = 0.62 for the intrapartum scale (*p* < 0.001), and *r* = 0.78 for the postnatal scale (*p* < 0.001).

### Responsiveness

Cut-off scores with the Receiver Operating Characteristic (ROC) on the optimum cut-off scores for antenatal, intrapartum and postnatal scores were equal to or higher than 48, 50 and 70, respectively, for optimal satisfaction. The scores for non-optimal satisfaction were below those numbers. The three scales show a good sensitivity and a very good specificity > 0.80%, except for the antenatal scale, with a specificity of 63%. The analysis to assess the efficacy of predictors with the AUC of the satisfaction score was adequate for the three scales (Table 2).

### Construct validity

No associations were shown with the linear regression between the score of satisfaction and the time taken to return the questionnaire for the three scales: antenatal scale, y = 48.65 + 0.01*x; *n* = 196; R2 = 0.01; *p* = 0.54, intrapartum scale: y = 65.14–0.01*x; *n* = 202; R2 = 0.01; *p* = 0.66 and postnatal scale, y = 71.17–0.009*x; *n* = 202; R2 = 0.001; *p* = 0.84.

### Internal consistency

The instrument shows an adequate internal consistency, which was evaluated using the Cronbach’s alpha coefficient; the results of each scale ranged from 0.85 to 0.95 and can be found in Table 7.

**Table 7:** Homogeneity of the interrelated items and scores for each scale

	Cronbach’s alpha^a^	Correlations inter-items scale^a^	Correlations between the items scale and the overall evaluation
Antenatal	0.85	0.34^c^ to 0.69^c^	0.73^c^
Intrapartum	0.90	0.35^c^ to 0.79^c^	0.73^c^
Postnatal	0.95	0.39^c^ to 0.90^c^	0.80^c^

^a^ Without the item of overall evaluation (last item of each scale)

^b^ Spearman’s rho correlation is significant at the 0.05 level (bilateral)

^c^ Spearman’s rho correlation is significant at the 0.01 level (bilateral)

The homogeneity of interrelated items on each scale was statistically significant, and when evaluated using the Spearman’s rho correlation coefficient, the results were moderate to strong. Thus, correlations between the sum of the items and the score of the overall evaluation on each scale were strong. The Spearman’s correlation coefficient reached *r* = 0.73 for the antenatal scale, *r* = 0.73 for the intrapartum scale and *r* = 0.80 for the postnatal scale (Table 7).

### Reliability and reproducibility

The mean of days between the questionnaire and the retest was 64.6 days (SD = 34.0). The reliability coefficients for the three scales were estimated using ICC and were found to be above 0.80. The absolute reliability was evaluated with the standard error of measurement showing a reasonable limit on the three scales, ranging from 2.9 to 6.3 (Table 8).

**Table 8:** Properties of the reproducibility of the ESEM instrument

ESEM Scale	Test Mean (SD)	Retest Mean (SD)	Mean Difference	ICC (95% IC)	SEM (SEM%)	SRD (SRD%)
Antenatal (*n* = 193)	49.6 (6.6)	48.4 (6.6)	−1.1 (4.1)	0.80 (0.75 and 0.85)	2.9 (5.9)	8.1 (16.3)
Intrapartum (*n* = 201)	64.2 (8.2)	62.9 (8.8)	−1.3 (4.1)	0.88 (0.85 and 0.91)	2.8 (4.4)	7.8 (12.3)
Postnatal (*n* = 199)	70.4 (14.4)	69.8 (13.7)	−0.9 (7.2)	0.81 (0.76 and 0.85)	6.3 (8.9)	17.2 (24.5)

*ICC* Intra-class correlation coefficient, *SEM* Standard error of measurement, *SRD* Smallest real difference

### Measure of agreement

**Fig. 2:**
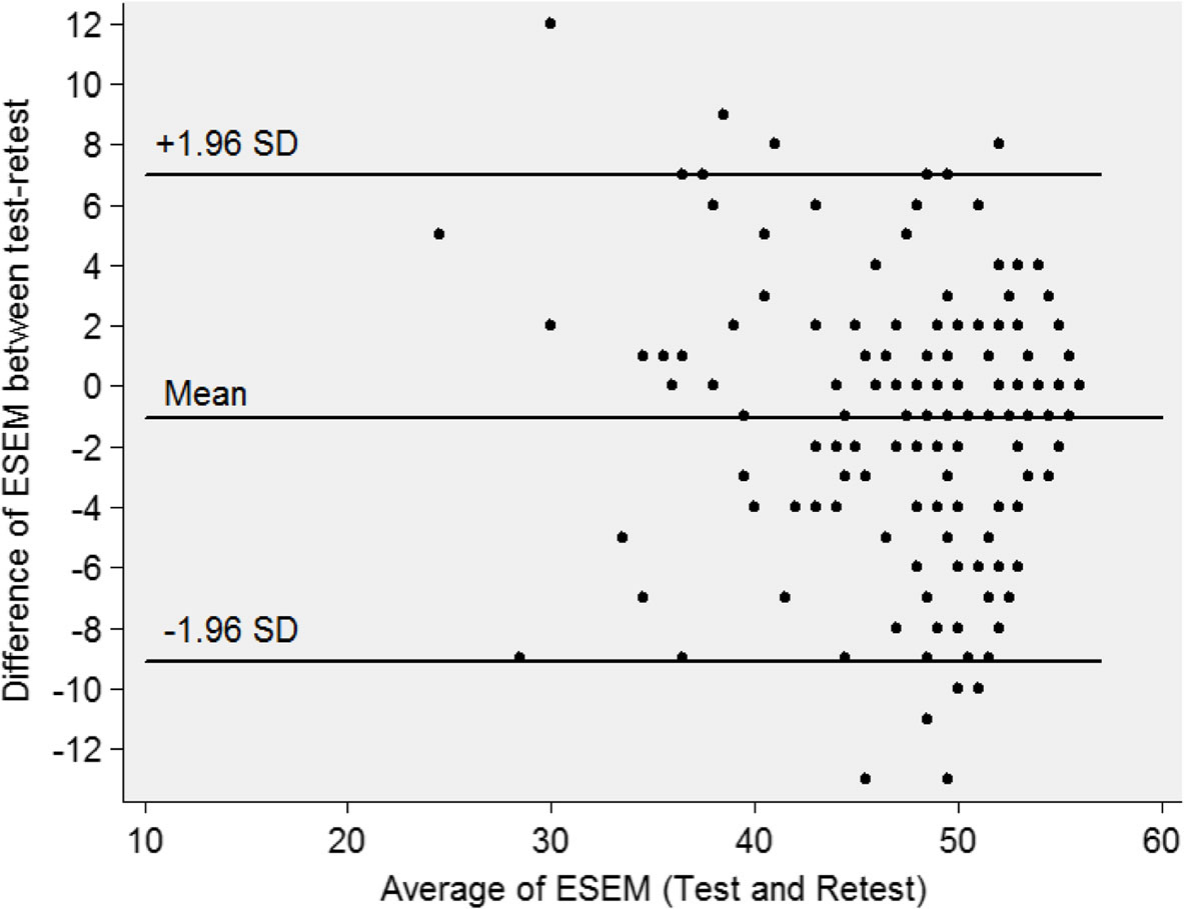
Bland-Altman Plots for antenatal scale

**Fig. 3:**
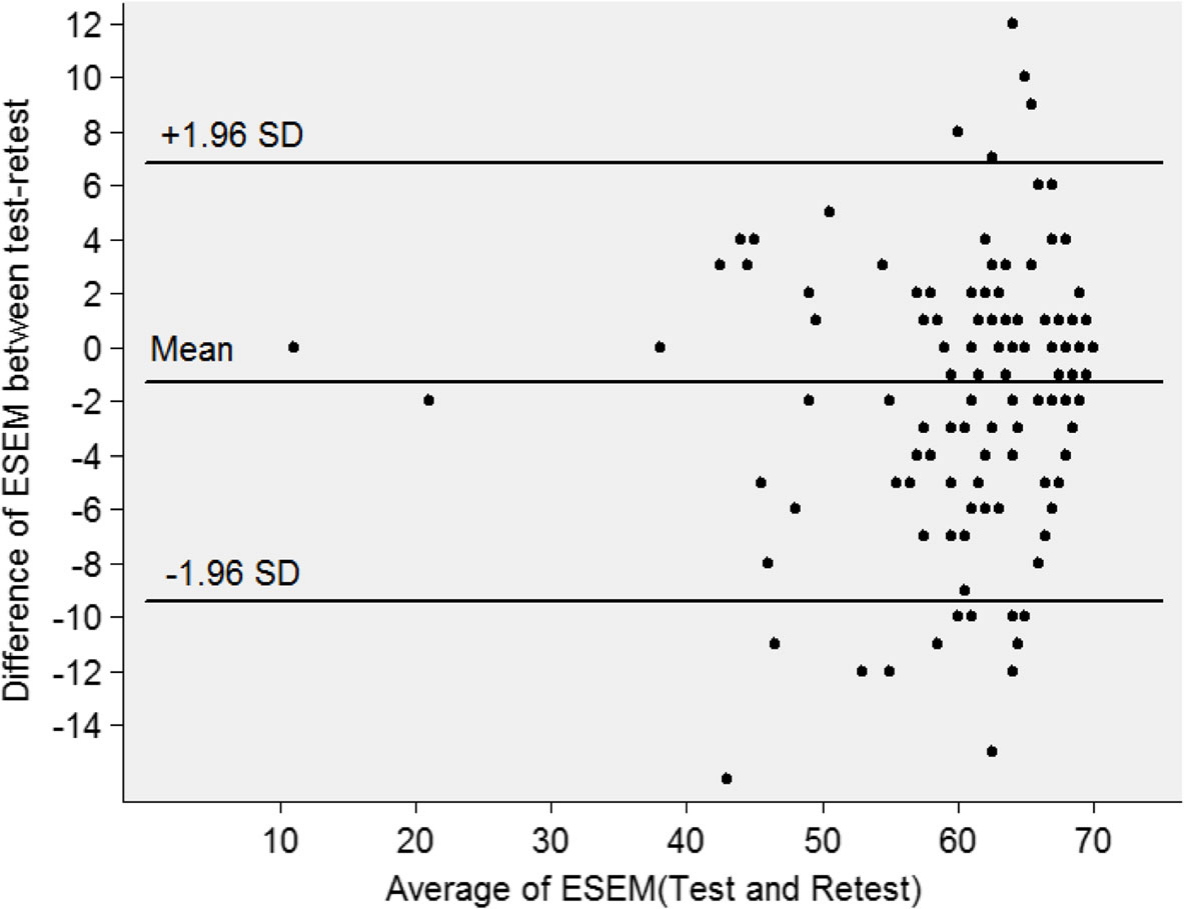
Bland-Altman Plots for intrapartum scale

**Fig. 4:**
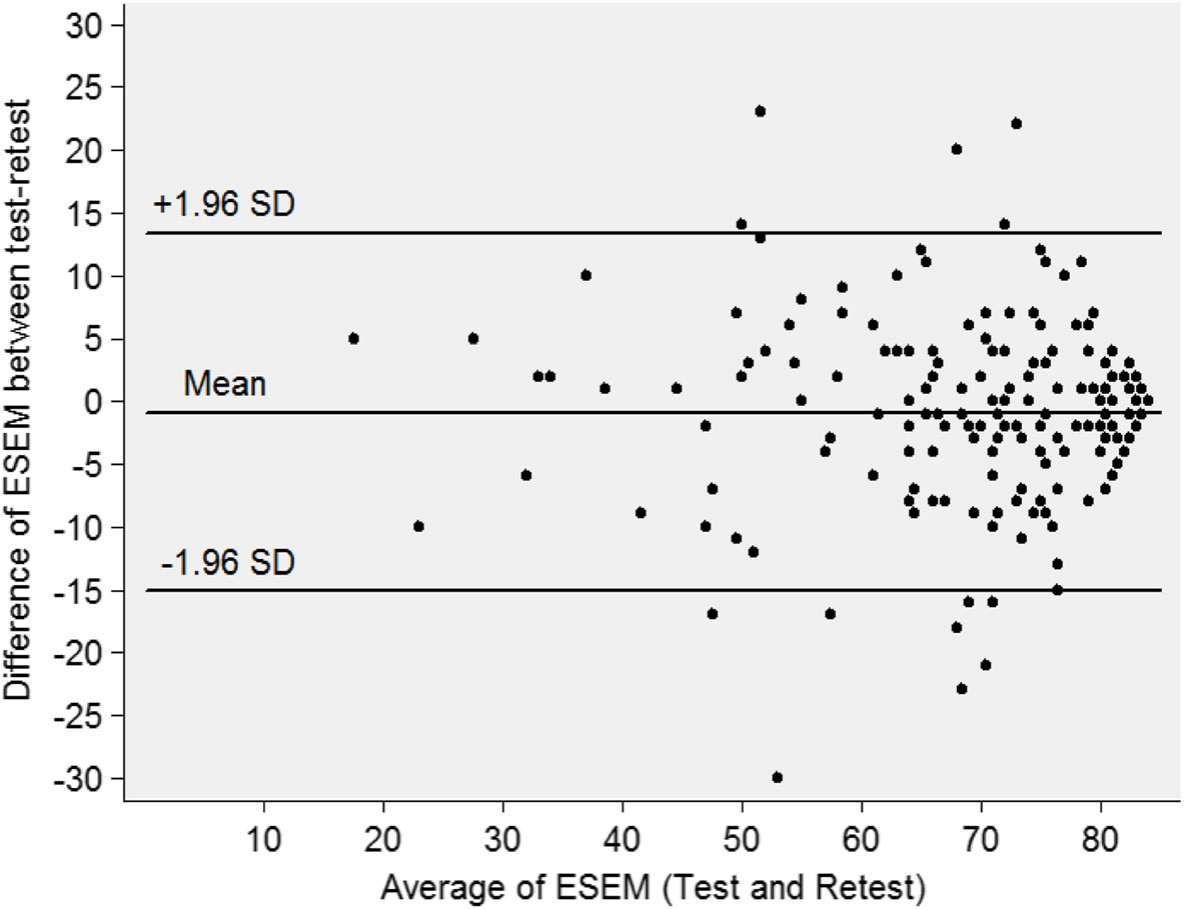
Bland-Altman Plots for postnatal scale

The Bland-Altman analysis shows that, for the antenatal scale, the mean of the difference was − 1.06 (Fig. 2). The 95% limit of agreement between the test and retest ranged from − 9.10 to 6.98, (93.3%). For the intrapartum scale, the mean of the difference was − 1.30, and the 95% limit of agreement between the test and retest ranged from − 9.41 to 6.81 (91.0%). For the postnatal scale, the mean of the difference was 0.86, and the 95% limit of agreement between the test and retest varied from − 15.05 to 15.05, showing 93.9% of the limit of the agreement (Figs. 3 and 4).

## Discussion

### Sample and recruitment procedure

In the present study, the ‘Women’s Experience of Maternity Care’ scale offers measurements of the level of satisfaction which are temporal to perinatal care and were taken into consideration for antenatal, intrapartum and postnatal care aspects. This study presented the results and detailed the processes of translating the questionnaire in French with cross-cultural adaptation of the WEMCS as well as discuss the preliminary psychometric properties of the instrument called the ‘Échelle de Satisfaction de l’Experience des Soins en Maternité’ (ESEM). This study also determined the value of each scale individually which can differentiate between satisfaction and dissatisfaction. The French version of this instrument had good acceptability, which was confirmed by the high response rate and, globally, few missing values.

### Instrument properties

We observed a high ceiling effect for the intrapartum scales; this problem was also observed in the original studies [20, 27]. If a 15% ceiling effect is suggested [37], many researchers have observed ceiling effects in their research and scales which have high rates of satisfaction [49]. A similar demonstration of the ceiling effect was shown in the maternity experience survey of the National Perinatal Epidemiology [50]. In the present study, the ceiling effects detected an inadequate measurement precision on the upper end of the scale [51]. This was confirmed in cognitive debriefing with the pre-test scale when participants explained that the maximum level item was not high enough, as the health care received was perfect. This problem demonstrates a lack of precision or detail in the satisfaction scales. The public target of a selection of mothers without obstetrical complications could have limited the results of the psychometric properties of the ESEM. The use of the Visual Analogue Scale could control for the ceiling effect [52]. Another element to consider is the relationship between the time the women completed the questionnaire and their degree of satisfaction. In our study, if the questionnaire was sent 2 months after birth, the mean of the return of the questionnaire by the mother was almost 90 days, with a large standard deviation. In our simple, we did not find any significant change in the women’s degree of satisfaction between the time of birth and the time of questionnaire completion, suggesting the Hawthorne effect [53, 54]. However, it is necessary to consider that a woman’s view about her childbirth experience changes over time [55, 56]. Waldenström suggested that having an emergency caesarean is the major determinant in the change from a very positive experience at 2 months after birth to a less positive experience at 1 year after birth [55]. Other factors that need to be emphasised are women’s perceptions of pain and/or socio-economic factors, such as being a single mother or experiencing health problems (e.g. anxiety); these elements could negatively influence childbirth satisfaction at 1 year compared with that at 2 months [50]. In the work of Waldenström (2004), the women who changed their perceptions of childbirth from a very negative one to a less negative one were those who received satisfactory support from midwives. In the literature, there is no consensus on when to measure satisfaction after birth [8]. Another aspect that we considered in the context of Switzerland is the difficulty of the mothers in answering the questionnaire at that particular time, which is likely connected to their intense emotional experiences during the post-partum period [39]. In our experience, although the mothers would often like to respond, they did not have the time to do so and there was frequently a long period of time between when the questionnaire was sent and when the women returned it, therefore a reminder was frequently necessary.

### Exploratory factor

The number of latent variables of each scale was examined with an exploration of the factor analysis. This exploration could confirm the unidimensionality of the three scales, with the highest percent of variance explained by one factor, which characterises the perception of the global experience of each period of perinatality. Factor loadings of each scale presented adequate results, except for the item negatively formulated, ‘The doctor and midwives were very rushed’. Reformulation of this item should be considered so that it is written in a way that does not create confusion. This result was confirmed by the result of communalities. The communalities could explain how much of the variance is captured by the items in this component. The findings of this study presented a satisfactory proportion of variables explained by the common factors, except for the item, ‘The doctor and midwives were very rushed’, on each scale.

### Responsiveness

The utilisation of the ESEM scale to test the satisfaction in dichotomised global scales was not presented in an anterior study. The best cut-off points used to evaluate the optimal satisfaction vs the non-optimal satisfaction of each scale show a good sensitivity and specificity for each period of care. The findings of this test present a valid interpretation of the dichotomised scores.

### Internal consistency could estimate the homogeneity of the items

The scale had good consistency based on Cronbach’s alpha coefficients for the three scales. The three scales showed the convergent construct validity of interrelated-item scales. The inter-total scale also had appropriate construct validity, with moderate and good correlation on the five-item scales. The convergent construct validity of the antenatal scale was slightly lower than that of the intrapartum and postnatal scales. The external validity compared with the original study was adequate with respect to the limit of the data available, the sample size of the present study and the different midwife team care offered in the respective studies [27].

The reproducibility of the tool evaluated by test-retest was satisfactory despite the time-lapse between the two evaluations. The time period which is generally accepted is 15 days [37], but we have previously observed that women experience some difficulty participating in studies immediately after giving birth as they have a lack of time [39]. Overall, the mean of the three scales was slightly lower than that of the overall test. However, this difference was not significant, and reliability was consistent between the test and retest. This element was confirmed by the ICC and a small error of measure between the two measures. The Bland-Altman plot demonstrated good agreement.

### Limitations

Our study had several limitations. Satisfaction is a subjective measure, limiting the interpretation of the results, particularly given that evaluation was conducted after a period of distance from the event. The structure of the scale with the majority of items were positive, and only one negatively expressed item was problematic to interpret properly the result. The lack of comparison with the original languages for psychometric properties limited the validation process of the scale. To perform confirmatory analysis, it would have been necessary to include an additional 300 participants, as it is not recommended to conduct confirmatory and exploratory analysis using the same sample [57, 58]. Time constraints did not allow doubling the study sample, so we chose to review the literature on recommendations to validate an instrument that has never been tested and to proceed first with the exploratory analysis [33]. The selection bias of the participants is also a limitation, as the participants in this study were not randomised. We also cannot take into account problems stemming from ceiling and floor effects, which limited our ability to show the level of detail of satisfaction. Therefore, this toll must be tested in the context of different models of women’s care and with women with differing degrees of pregnancy complications.

### Strengths

The ESEM focusses on the actual experiences of women with maternity care, while existing questionnaires focus strictly on a specific stage of the maternity pathway [7]. To prevent crossover effects from postnatal care to labour experience or from labour and birth experience to antenatal experience, the three separate questionnaires measure experiences during prenatal care, delivery and postpartum care distinctly. By covering the three periods of maternity care, the ESEM will facilitate quality improvement, as the services usually involved are distinctly different. Another strength of the research is the low attrition of less than 10%, which is much lower than the normal attrition of similar validation studies (e.g., 24% in [59] and 41% in [60]).

## Conclusion

Measuring the satisfaction of women across the spectrum of maternity care episodes, from the first antenatal visit to the last day in the postnatal ward, is essential to have a clear idea of women’s journeys and related satisfaction across the different episodes of care. To our knowledge, this is the first French-language scale which assessed the degree of satisfaction during the perinatal period. We encourage other researchers in French-speaking countries to use this tool, as it provides a measurement not only of overall satisfaction, but also of satisfaction level through the antenatal, intrapartum and postnatal care.

## Supplementary information

**Additional file 1.**

**Additional file 2.**

## Data Availability

The birth record data used in this study will not be shared because they contain confidential information, including the names of the mother and the dates of birth of both the mothers and infants.

## Abbreviations

ESEM: Échelle de Satisfaction de l’Experience des Soins en Maternité
WEMCS: Women’s Experiences Maternity Care Scale
LAS: Labour Agentry Scale
BSC: Birth Satisfaction Scale
CEQ: Childbirth Experiences Questionnaire
WOMBLSQ4: Women’s Views of Birth Labour Satisfaction Questionnaire 4
EPDS: Edinburgh Postnatal Depression Scale
HUG: University Hospital of Geneva
