# Supplementary material for: Cross-cultural validation and psychometrics’ evaluation of women’s experience of maternity care scale in French: the ESEM
Source: BMC Med Res Methodol. 2020 Jul 11;20:188. doi: 10.1186/s12874-020-01052-5 (PMC7353730; doi:10.1186/s12874-020-01052-5)
Supplement: Supplementary file 1 — Additional file 1. [file 12874_2020_1052_MOESM1_ESM.docx]

**Women’s experience of maternity care^1^**

^1^The original English questionnaire, ‘Women’s experience of maternity care’, is the result of various

change times. (Biro, Waldenström, Brown, & Pannifex, 2003; Brown & Lumley, 1994, 1998; Waldenström, 1999)

Some of the things that women have said about their care in pregnancy are listed below. We would like to know whether you would say the same things about your care.

Some questions are about the care provided by doctors and midwives. This care might have differed between the individual care providers, but please try to make *a comprehensive* assessment of the caregivers during your pregnancy.

**A. So thinking about the care you had while you were pregnant, please circle ONE number on EACH line to show whether you agree or disagree**

1. **At my check-ups I was always asked whether I had any questions**

| Disagree strongly | 1 | 2 | 3 | 4 | 5 | 6 | 7 | Agree strongly |
| --- | --- | --- | --- | --- | --- | --- | --- | --- |

1. **Often at my check-ups the doctors or midwives were very rushed**

| Disagree strongly | 1 | 2 | 3 | 4 | 5 | 6 | 7 | Agree strongly |
| --- | --- | --- | --- | --- | --- | --- | --- | --- |

1. **I always felt my worries, anxieties or concerns about the pregnancy and the baby were taken seriously by the doctors/midwives**

| Disagree strongly | 1 | 2 | 3 | 4 | 5 | 6 | 7 | Agree strongly |
| --- | --- | --- | --- | --- | --- | --- | --- | --- |

1. **I was always kept informed about what was happening and doctors/midwives made an effort to explain anything I didn’t understand**

| Disagree strongly | 1 | 2 | 3 | 4 | 5 | 6 | 7 | Agree strongly |
| --- | --- | --- | --- | --- | --- | --- | --- | --- |

1. **I was happy with the physical care I received in pregnancy from doctors/midwives**

| Disagree strongly | 1 | 2 | 3 | 4 | 5 | 6 | 7 | Agree strongly |
| --- | --- | --- | --- | --- | --- | --- | --- | --- |

1. **I was happy with the emotional support I received in pregnancy from doctors/midwives**

| Disagree strongly | 1 | 2 | 3 | 4 | 5 | 6 | 7 | Agree strongly |
| --- | --- | --- | --- | --- | --- | --- | --- | --- |

1. **I was always given an active say in decisions about my care in pregnancy**

| Disagree  strongly | 1 | 2 | 3 | 4 | 5 | 6 | 7 | Agree  strongly |
| --- | --- | --- | --- | --- | --- | --- | --- | --- |

1. **On balance how would you describe your CARE during pregnancy?**

| Very poor | 1 | 2 | 3 | 4 | 5 | 6 | 7 | Very good |
| --- | --- | --- | --- | --- | --- | --- | --- | --- |

**B. Giving birth is usually an overwhelming experience, physical as well emotionally. How would you describe your experience of giving birth ?**

**Please circle ONE number on EACH line to show whether you agree or disagree**

**1. The midwives and doctors always kept me informed about what was happening and made an effort to explain anything I didn’t understand**

| Disagree  strongly | 1 | 2 | 3 | 4 | 5 | 6 | 7 | Agree  strongly |
| --- | --- | --- | --- | --- | --- | --- | --- | --- |

**2. I was always given an active say in decisions about care during labour and birth**

| Disagree  strongly | 1 | 2 | 3 | 4 | 5 | 6 | 7 | Agree  strongly |
| --- | --- | --- | --- | --- | --- | --- | --- | --- |

**3. The doctors/midwives were sensitive and understanding**

| Disagree  strongly | 1 | 2 | 3 | 4 | 5 | 6 | 7 | Agree  strongly |
| --- | --- | --- | --- | --- | --- | --- | --- | --- |

**4. The doctors/midwives were encouraging and reassuring**

| Disagree  strongly | 1 | 2 | 3 | 4 | 5 | 6 | 7 | Agree  strongly |
| --- | --- | --- | --- | --- | --- | --- | --- | --- |

**5. I often felt the doctors/midwives were very rushed**

| Disagree  strongly | 1 | 2 | 3 | 4 | 5 | 6 | 7 | Agree  strongly |
| --- | --- | --- | --- | --- | --- | --- | --- | --- |

**6. Care during labour and birth was provided in a safe and competent way**

| Disagree  strongly | 1 | 2 | 3 | 4 | 5 | 6 | 7 | Agree  strongly |
| --- | --- | --- | --- | --- | --- | --- | --- | --- |

**7. I was happy with the physical aspect of care by doctors/midwives**

| Disagree  strongly | 1 | 2 | 3 | 4 | 5 | 6 | 7 | Agree  strongly |
| --- | --- | --- | --- | --- | --- | --- | --- | --- |

**8. I was happy with the emotional support I received by doctors/midwives**

| Disagree  strongly | 1 | 2 | 3 | 4 | 5 | 6 | 7 | Agree  strongly |
| --- | --- | --- | --- | --- | --- | --- | --- | --- |

**9. My needs of privacy were well respected during the labour and birth**

| Disagree  strongly | 1 | 2 | 3 | 4 | 5 | 6 | 7 | Agree  strongly |
| --- | --- | --- | --- | --- | --- | --- | --- | --- |

**10. On balance how would you describe your CARE in labour in labour and birth ?**

| Very poor | 1 | 2 | 3 | 4 | 5 | 6 | 7 | Very good |
| --- | --- | --- | --- | --- | --- | --- | --- | --- |

**C. What was your experience of CARE in hospital after birth.**

**Please circle ONE number on EACH line to show whether you agree or disagree**

**1. I was always kept informed about what was happening and doctors/midwives made an effort**

**to explain anything I didn’t understand**

| Disagree  strongly | 1 | 2 | 3 | 4 | 5 | 6 | 7 | Agree  strongly |
| --- | --- | --- | --- | --- | --- | --- | --- | --- |

**2. I was always given an active say in decisions about the care of my baby and myself**

| Disagree  strongly | 1 | 2 | 3 | 4 | 5 | 6 | 7 | Agree  strongly |
| --- | --- | --- | --- | --- | --- | --- | --- | --- |

**3. I was given the advice and support I needed in how to handle, settle or look after the baby**

| Disagree  strongly | 1 | 2 | 3 | 4 | 5 | 6 | 7 | Agree  strongly |
| --- | --- | --- | --- | --- | --- | --- | --- | --- |

**4. I was given the advice and support I needed in any problems with the baby’s health and**

**Progress**

| Disagree  strongly | 1 | 2 | 3 | 4 | 5 | 6 | 7 | Agree  strongly |
| --- | --- | --- | --- | --- | --- | --- | --- | --- |

**5. I was given the advice and support I needed about my own health and recovery**

| Disagree  strongly | 1 | 2 | 3 | 4 | 5 | 6 | 7 | Agree  strongly |
| --- | --- | --- | --- | --- | --- | --- | --- | --- |

**6.The midwives/doctors were sensitive and understanding**

| Disagree  strongly | 1 | 2 | 3 | 4 | 5 | 6 | 7 | Agree  strongly |
| --- | --- | --- | --- | --- | --- | --- | --- | --- |

**7. The doctors/midwives were encouraging and reassuring**

| Disagree  strongly | 1 | 2 | 3 | 4 | 5 | 6 | 7 | Agree  strongly |
| --- | --- | --- | --- | --- | --- | --- | --- | --- |

**8. I often felt the doctors/midwives were very rushed**

| Disagree  strongly | 1 | 2 | 3 | 4 | 5 | 6 | 7 | Agree  strongly |
| --- | --- | --- | --- | --- | --- | --- | --- | --- |

**9. Care in hospital after the birth was provided in a safe and competent way**

| Disagree  strongly | 1 | 2 | 3 | 4 | 5 | 6 | 7 | Agree  strongly |
| --- | --- | --- | --- | --- | --- | --- | --- | --- |

**10. I was happy with the physical aspect of care by doctors/midwives**

| Disagree strongly | 1 | 2 | 3 | 4 | 5 | 6 | 7 | Agree strongly |
| --- | --- | --- | --- | --- | --- | --- | --- | --- |

**11. I was happy with the emotional support I received by doctors/midwives**

| Disagree strongly | 1 | 2 | 3 | 4 | 5 | 6 | 7 | Agree strongly |
| --- | --- | --- | --- | --- | --- | --- | --- | --- |

**12.Thinking back now, how would you describe the care you and your baby received in**

hospital after the birth ?

| Very poor | 1 | 2 | 3 | 4 | 5 | 6 | 7 | Very good |
| --- | --- | --- | --- | --- | --- | --- | --- | --- |

Biro, M. A., Waldenström, U., Brown, S., & Pannifex, J. H. (2003). Satisfaction with team midwifery care for low- and high-risk women: a randomized controlled trial. *Birth, 30*(1), 1-10.

Brown, S., & Lumley, J. (1994). Satisfaction with care in labor and birth: a survey of 790 Australian women. *Birth, 21*(1), 4-13.

Brown, S., & Lumley, J. (1998). Changing childbirth: lessons from an Australian survey of 1336 women. *Br J Obstet Gynaecol, 105*(2), 143-155.

Waldenström, U. (1999). Experience of labor and birth in 1111 women. *J Psychosom Res, 47*(5), 471-482.
