# Supplementary material for: Cross-cultural validation and psychometrics’ evaluation of women’s experience of maternity care scale in French: the ESEM
Source: BMC Med Res Methodol. 2020 Jul 11;20:188. doi: 10.1186/s12874-020-01052-5 (PMC7353730; doi:10.1186/s12874-020-01052-5)
Supplement: Supplementary file 2 — Additional file 2. [file 12874_2020_1052_MOESM2_ESM.docx]

### **Appendix 2**

Échelle de Satisfaction de l’Expérience des Soins en Maternité (ESEM)^1^

(Women’s experience of maternity care)

***Soins pendant la grossesse***

Certains points soulignés par les femmes à propos de leur grossesse sont cités ci-dessous. Nous souhaiterions savoir si vous partagez la même opinion à propos des soins que vous avez reçus.

Certaines questions concernent les soins dispensés par les médecins et les sages-femmes.

Ces soins peuvent varier entre ces différents soignants, mais nous vous prions de les évaluer de manière globale.

Nous vous demandons donc de penser aux soins reçus pendant **votre grossesse** et d'entourer UN SEUL numéro pour chaque phrase pour indiquer dans quelle mesure vous êtes d'accord ou non.

1. **Lors des visites de contrôle, on m'a toujours demandé si j'avais des questions**

| Pas du tout d'accord | 1 | 2 | 3 | 4 | 5 | 6 | 7 | Tout à fait d'accord |
| --- | --- | --- | --- | --- | --- | --- | --- | --- |

1. **Souvent lors de mes contrôles, les médecins et les sages-femmes étaient très pressés**

| Pas du tout d'accord | 1 | 2 | 3 | 4 | 5 | 6 | 7 | Tout à fait d'accord |
| --- | --- | --- | --- | --- | --- | --- | --- | --- |

1. **J’ai toujours eu l’impression que mes soucis, angoisses ou préoccupations à propos de**

**la grossesse et du bébé étaient pris au sérieux par les médecins et les sages-femmes**

| Pas du tout d'accord | 1 | 2 | 3 | 4 | 5 | 6 | 7 | Tout à fait d'accord |
| --- | --- | --- | --- | --- | --- | --- | --- | --- |

1. **J’ai toujours été tenue informée de ce qui se passait et les médecins et les**

**sages-femmes se sont efforcés de m’expliquer ce que je ne comprenais pas**

| Pas du tout d'accord | 1 | 2 | 3 | 4 | 5 | 6 | 7 | Tout à fait d'accord |
| --- | --- | --- | --- | --- | --- | --- | --- | --- |

1. **J’ai été satisfaite des soins reçus de la part des médecins et des sages-femmes**

**pendant ma grossesse**

| Pas du tout d'accord | 1 | 2 | 3 | 4 | 5 | 6 | 7 | Tout à fait d'accord |
| --- | --- | --- | --- | --- | --- | --- | --- | --- |

1. **J’ai été satisfaite du soutien moral (émotionnel) reçu de la part des médecins et**

**des sages-femmes pendant ma grossesse**

| Pas du tout d'accord | 1 | 2 | 3 | 4 | 5 | 6 | 7 | Tout à fait d'accord |
| --- | --- | --- | --- | --- | --- | --- | --- | --- |

1. **J’ai toujours eu mon mot à dire dans les décisions concernant les soins reçus pendant**

**ma grossesse**

| Pas du tout d'accord | 1 | 2 | 3 | 4 | 5 | 6 | 7 | Tout à fait d'accord |
| --- | --- | --- | --- | --- | --- | --- | --- | --- |

1. **Sur l’échelle suivante, comment décririez-vous les SOINS reçus pendant votre grossesse ?**

| Très mauvais | 1 | 2 | 3 | 4 | 5 | 6 | 7 | Excellents |
| --- | --- | --- | --- | --- | --- | --- | --- | --- |

***Soins pendant le travail et l’accouchement***

En général, donner naissance est une expérience très forte, physiquement autant que moralement (émotionnellement). Comment décririez-vous votre propre vécu de l’accouchement ?

Certaines questions concernent les soins dispensés par les médecins et les sages-femmes. Ces soins peuvent varier entre ces différents soignants, mais nous vous prions de les évaluer de manière globale.

Nous vous demandons donc de penser aux soins reçus pendant **le travail et l’accouchement** et d'entourer **UN SEUL** numéro pour chaque phrase pour indiquer dans quelle mesure vous êtes d'accord ou non.

1. **Les médecins et les sages-femmes m’ont toujours tenue informée de**

**ce qui se passait et se sont efforcés de m’expliquer ce que je ne comprenais pas**

| Pas du tout d'accord | 1 | 2 | 3 | 4 | 5 | 6 | 7 | Tout à fait d'accord |
| --- | --- | --- | --- | --- | --- | --- | --- | --- |

1. **J’ai toujours eu mon mot à dire à propos des décisions sur les soins dispensés**

**lors du travail et de l’accouchement**

| Pas du tout d'accord | 1 | 2 | 3 | 4 | 5 | 6 | 7 | Tout à fait d'accord |
| --- | --- | --- | --- | --- | --- | --- | --- | --- |

1. **Les médecins et les sages-femmes étaient sensibles et compréhensifs**

| Pas du tout d'accord | 1 | 2 | 3 | 4 | 5 | 6 | 7 | Tout à fait d'accord |
| --- | --- | --- | --- | --- | --- | --- | --- | --- |

1. **Les médecins et les sages-femmes étaient encourageants et rassurants**

| Pas du tout d'accord | 1 | 2 | 3 | 4 | 5 | 6 | 7 | Tout à fait d'accord |
| --- | --- | --- | --- | --- | --- | --- | --- | --- |

1. **J’ai souvent eu l’impression que les médecins et les sages-femmes étaient très pressés**

| Pas du tout d'accord | 1 | 2 | 3 | 4 | 5 | 6 | 7 | Tout à fait d'accord |
| --- | --- | --- | --- | --- | --- | --- | --- | --- |

1. **Lors du travail et de l’accouchement, les soins ont été dispensés avec assurance**

**et compétence**

| Pas du tout d'accord | 1 | 2 | 3 | 4 | 5 | 6 | 7 | Tout à fait d'accord |
| --- | --- | --- | --- | --- | --- | --- | --- | --- |

1. **J’ai été satisfaite des soins dispensés par les médecins et les sages-femmes**

| Pas du tout d'accord | 1 | 2 | 3 | 4 | 5 | 6 | 7 | Tout à fait d'accord |
| --- | --- | --- | --- | --- | --- | --- | --- | --- |

1. **J’ai été satisfaite du soutien émotionnel (moral) dispensé par les médecins**

**et les sages-femmes**

| Pas du tout d'accord | 1 | 2 | 3 | 4 | 5 | 6 | 7 | Tout à fait d'accord |
| --- | --- | --- | --- | --- | --- | --- | --- | --- |

1. **Mes besoins d’intimité ont été bien respectés pendant le travail et l’accouchement**

| Pas du tout d'accord | 1 | 2 | 3 | 4 | 5 | 6 | 7 | Tout à fait d'accord |
| --- | --- | --- | --- | --- | --- | --- | --- | --- |

**10. Sur l’échelle suivante, comment décririez-vous les SOINS reçus lors du travail et**

**de l’accouchement ?**

| Très mauvais | 1 | 2 | 3 | 4 | 5 | 6 | 7 | Excellents |
| --- | --- | --- | --- | --- | --- | --- | --- | --- |

***Soins pendant le séjour à l’hôpital***

Certains points soulignés par les femmes à propos des soins reçus après leur accouchement, sont cités ci-dessous. Nous souhaiterions savoir si vous partagez la même opinion à propos des soins reçus.

Certaines questions concernent les soins dispensés par les médecins et les sages-femmes. Ces soins peuvent varier entre ces différents soignants, mais nous vous prions de les évaluer de manière globale.

Nous vous demandons donc de penser aux soins reçus **après l’accouchement**, **lors de votre séjour à l’hôpital** et d'entourer UN SEUL numéro pour chaque phrase pour indiquer dans quelle mesure vous êtes d'accord ou non.

**1. Les médecins et les sages-femmes m’ont toujours tenue informée de ce qui se passait**

**et se sont efforcés de m’expliquer ce que je ne comprenais pas**

| Pas du tout d'accord | 1 | 2 | 3 | 4 | 5 | 6 | 7 | Tout à fait d'accord |
| --- | --- | --- | --- | --- | --- | --- | --- | --- |

**2. J’ai toujours eu mon mot à dire à propos des décisions sur les soins dispensés**

**à mon bébé et à moi-même**

| Pas du tout d'accord | 1 | 2 | 3 | 4 | 5 | 6 | 7 | Tout à fait d'accord |
| --- | --- | --- | --- | --- | --- | --- | --- | --- |

**3**. **J’ai reçu les conseils et le soutien dont j’avais besoin pour porter le bébé, le calmer ou**

**m’occuper de lui**

| Pas du tout d'accord | 1 | 2 | 3 | 4 | 5 | 6 | 7 | Tout à fait d'accord |
| --- | --- | --- | --- | --- | --- | --- | --- | --- |

**4. J’ai reçu les conseils et le soutien dont j’avais besoin en cas de problème lié à la santé**

**et au développement de mon bébé**

| Pas du tout d'accord | 1 | 2 | 3 | 4 | 5 | 6 | 7 | Tout à fait d'accord |
| --- | --- | --- | --- | --- | --- | --- | --- | --- |

**5. J’ai reçu les conseils et le soutien dont j’avais besoin pour ma santé et mon**

**rétablissement**

| Pas du tout d'accord | 1 | 2 | 3 | 4 | 5 | 6 | 7 | Tout à fait d'accord |
| --- | --- | --- | --- | --- | --- | --- | --- | --- |

**6. Les médecins et les sages-femmes étaient sensibles et compréhensifs**

| Pas du tout d'accord | 1 | 2 | 3 | 4 | 5 | 6 | 7 | Tout à fait d'accord |
| --- | --- | --- | --- | --- | --- | --- | --- | --- |

**7. Les médecins et les sages-femmes étaient encourageants et rassurants**

| Pas du tout d'accord | 1 | 2 | 3 | 4 | 5 | 6 | 7 | Tout à fait d'accord |
| --- | --- | --- | --- | --- | --- | --- | --- | --- |

**8 J’ai souvent eu l’impression que les médecins et les sages-femmes étaient très pressés**

| Pas du tout d'accord | 1 | 2 | 3 | 4 | 5 | 6 | 7 | Tout à fait d'accord |
| --- | --- | --- | --- | --- | --- | --- | --- | --- |

**9. Après l’accouchement les soins à l’hôpital ont été dispensés avec assurance et**

**compétence**

| Pas du tout d'accord | 1 | 2 | 3 | 4 | 5 | 6 | 7 | Tout à fait d'accord |
| --- | --- | --- | --- | --- | --- | --- | --- | --- |

**10. J’ai été satisfaite des soins** **dispensés par les médecins et les sages-femmes**

| Pas du tout d'accord | 1 | 2 | 3 | 4 | 5 | 6 | 7 | Tout à fait d'accord |
| --- | --- | --- | --- | --- | --- | --- | --- | --- |

**11. J’ai été satisfaite du soutien émotionnel (moral) dispensé par les médecins**

**et les sages femmes**

| Pas du tout d'accord | 1 | 2 | 3 | 4 | 5 | 6 | 7 | Tout à fait d'accord |
| --- | --- | --- | --- | --- | --- | --- | --- | --- |

**12. Lorsque vous y repensez, comment décririez-vous les SOINS que votre bébé et vous-même**

**avez reçus à l’hôpital après l’accouchement**

| Très mauvais | 1 | 2 | 3 | 4 | 5 | 6 | 7 | Excellents |
| --- | --- | --- | --- | --- | --- | --- | --- | --- |

^1^ Le questionnaire original a été conçu et adapté par différents auteurs : Biro, Waldenström, Brown, & Pannifex, 2003; Brown & Lumley, 1994, 1998; Waldenström, 1999.

Références

Biro, M. A., Waldenström, U., Brown, S., & Pannifex, J. H. (2003). Satisfaction with team midwifery care for low- and high-risk women: a randomized controlled trial. Birth, 30(1), 1-10.

Brown, S., & Lumley, J. (1994). Satisfaction with care in labor and birth: a survey of 790 Australian women. Birth, 21(1), 4-13.

Brown, S., & Lumley, J. (1998). Changing childbirth: lessons from an Australian survey of 1336 women. Br J Obstet Gynaecol, 105(2), 143-155.

Waldenström, U. (1999). Experience of labor and birth in 1111 women. J Psychosom Res, 47(5), 471-482.
